# Explainable, modality-adaptive radiomics for MGMT methylation prediction in high-grade glioma: a decision-curve analysis study

**DOI:** 10.3389/fonc.2025.1731258

**Published:** 2026-01-09

**Authors:** Rafail C. Christodoulou, Georgios Vamvouras, Rafael Pitsillos, Elena E. Solomou, Michalis F. Georgiou

**Affiliations:** 1 Department of Radiology, Stanford University School of Medicine, Stanford, CA, United States; 2 Department of Mechanical Engineering, National Technical University of Athens, Zografou, Greece; 3 Neurophysiology Department, Cyprus Institute of Neurology and Genetics, Nicosia, Cyprus; 4 Internal Medicine-Hematology, University of Patras Medical School, Rion, Greece; 5 Department of Radiology, Division of Nuclear Medicine, University of Miami, Miami, FL, United States

**Keywords:** decision curve analysis, explainable AI, HGG = high-grade glioma, MGMT = [6]-methylguanine-DNA methyltransferase, radiomics

## Abstract

**Introduction:**

MGMT promoter methylation is a critical predictive biomarker in high-grade gliomas (HGG), but its assessment currently relies on invasive tissue sampling. We aimed to develop an explainable, modality-adaptive, and calibrated radiomics model for non-invasive prediction of MGMT promoter methylation using multi-center MRI data.

**Methods:**

Pre-operative MRI from the UCSF-PDGM and UPENN-GBM cohorts was analyzed using radiomics extracted from intratumoral and peritumoral regions. Conventional (T1, T2, FLAIR) and advanced (DWI/ADC, ASL) MRI sequences were included. A novel modality-adaptive framework was implemented, allowing the model to automatically ignore advanced modalities when unavailable. After feature ranking and redundancy reduction, six machine-learning classifiers were optimized and Platt-calibrated. Model performance was evaluated on a held-out test set using ROC metrics, calibration assessment, and Decision Curve Analysis (DCA). Feature contributions were interpreted using SHAP.

**Results:**

The top-performing LightGBM model, trained on the 500 most important radiomic features, achieved an AUC of 0.67, recall of 0.90, and accuracy of 0.72 on the independent test set. The model demonstrated strong sensitivity for identifying methylated tumors, minimizing false-negative predictions. Calibration improved clinical net benefit across a range of threshold probabilities on DCA. Feature attribution analysis revealed balanced contributions from conventional and advanced MRI modalities, with texture and intensity-based descriptors being most influential. Notably, low-frequency FLAIR wavelet intensity features were associated with unmethylated tumors.

**Conclusion:**

This explainable, modality-adaptive radiomics model identified biologically consistent MGMT-related imaging patterns and demonstrated decision-analytic value for clinical risk stratification. The framework supports real-world applicability in heterogeneous imaging environments. Future work should focus on external validation and integration with clinical and molecular biomarkers to further enhance predictive performance.

## Graphical Abstract

**Figure:**
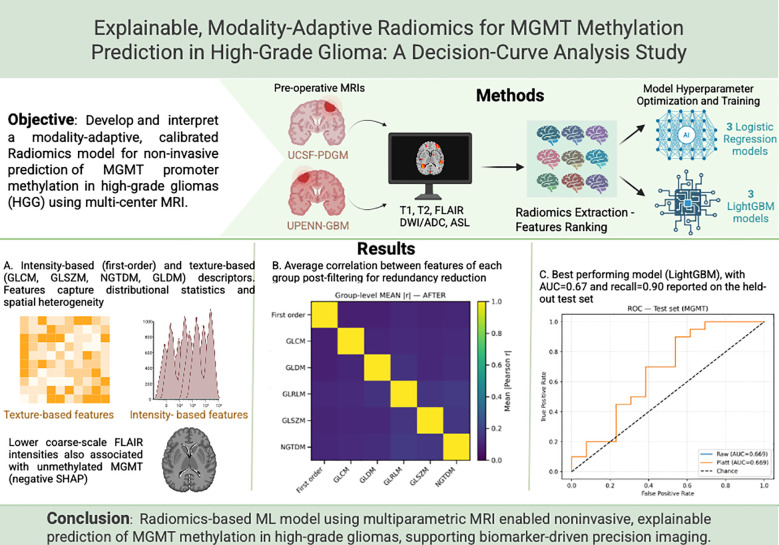

## Introduction

High-grade gliomas (HGG) are the most common and aggressive type of primary brain tumors and are characterized by poor prognosis and treatment resistance. The DNA-repair enzyme O^6-methylguanine-DNA methyltransferase (MGMT) plays a central role in resistance to alkylating agents since it opposes their antitumoral effects (1, 2). Epigenetic silencing via methylation of the MGMT gene promoter is crucial in predicting patient prognosis, as it reduces the enzyme’s repair ability and increases responsiveness to temozolomide chemotherapy (3). Hence, MGMT promoter methylation can serve as a predictive and prognostic biomarker guiding the selection of temozolomide-based regimens (4). Assessing MGMT status involves invasive tissue sampling, which carries surgical risks and can be confounded by intratumoral heterogeneity, particularly in glioblastoma (GBM).

MRI is considered the gold standard in glioma diagnosis. It can noninvasively provide information about tumor heterogeneity, predicting the genomic profile, treatment response, and prognosis. MGMT methylation correlates with distinct imaging features, including tumor location and necrosis (5). Advanced MRI sequences, such as ADC(cellularity) and ASL(perfusion), correlated with methylation status (6). Radiomics involves the high-throughput extraction of quantitative imaging features that measure tumor intensity, shape, texture, and spatial heterogeneity, turning standard medical images into a large volume of data (7, 8). When analyzed with machine learning algorithms, these features can identify subtle imaging biomarkers that reflect gliomas’ underlying histopathologic and molecular traits. In this setting, radiomic models have been widely studied for predicting noninvasively significant genetic changes, MGMT methylation, IDH mutation, and 1p/19q co-deletion (9).

A recent meta-analysis of 26 radiomics studies on MGMT highlights the growing interest in this area. It found that pooled AUC performance in grade IV gliomas varies from 0.57 to 0.73, but only a few studies included calibration and decision curve analysis. The analysis also concluded that MGMT appearance can be variable and that radiomics features are not yet robust enough to predict methylation status before surgery (10). These results highlight the urgent need for more robust and interpretable models to generalize across diverse imaging data. Existing studies often rely on single-center data and lack probability calibration and explainability, which restricts clinical adaptability (5, 10). In addition, most studies have focused exclusively on the intratumoral area despite growing evidence that the pre-tumoral microenvironment carries valuable information reflecting tumor infiltration, edema, and microvascular remodeling related to MGMT promoter methylation (11).

We developed an explainable multimodal radiomics framework for the non-invasive prediction of MGMT status in HGG. The pipeline uses imaging data from multiple institutions and combines intratumoral and peritumoral regions, capturing spatial information across various MRI modalities. Advanced MRI techniques, such as SWI/ADC, detect cellularity differences related to MGMT status, while ASL assesses tumor perfusion differences. The model dynamically integrates advanced MRI sequences such as ADC and ASL when present and automatically adapts when these are unavailable through modality-aware feature imputation. This design allows the algorithm to learn to ignore missing modalities while leveraging all available information, reflecting the variability encountered in real-world clinical practice and enhancing generalizability across sites. High-level preprocessing, redundancy reduction, and feature importance ranking were implemented, followed by the training of optimized ML classifiers. To enhance transparency and clinical adaptability, SHAP values were employed to quantify the contribution of individual radiomic features to the prediction. Probability calibration was implemented in the pipeline to ensure reliable and clinically meaningful prediction probabilities, given the crucial role of MGMT in treatment selection. Finally, to assess the clinical benefit of the proposed model, a decision curve analysis (DCA) was performed, quantifying the net benefit of model-assisted decisions across different probability thresholds compared with standard “treat-all” or “treat-none” strategies. Therefore, we aim to develop a reproducible, calibrated, and interpretable multiregional radiomics model that can be a valuable tool for predicting MGMT status in pre-operative glioma patients.

## Methodology

### MRI data acquisition

The MRI scans were obtained from the UCSF-PDGM (TCIA) and UPENN-GBM datasets (12, 13).

For each patient within the UCSF-PDGM dataset, the 3D sequences received are 3D T2-weighted, T2/FLAIR-weighted, susceptibility-weighted (SWI), diffusion-weighted (DWI), pre- and post-contrast T1-weighted images, and 3D arterial spin labelling (ASL) perfusion images. Most scans used a 3.0 Tesla scanner and two gadolinium-based contrast agents. Multicompartment tumor segmentation in the dataset was performed as part of the 2021 BraTS challenge. An ensemble of top-performing BraTS algorithms was first used for automated segmentation, followed by manual corrections by trained radiologists and final approval by two expert neuroradiologists. The resulting annotations include three main tumor compartments: enhancing tumor, non-enhancing or necrotic core, and surrounding FLAIR hyperintensity.

Within the UPENN-GBM dataset, for each subject, pre-operative and in some cases follow-up multimodal MRI scans are provided, including T1-weighted, post-contrast T1-weighted (T1-Gd), T2-weighted, and T2-FLAIR images, with most cases also containing diffusion tensor imaging (DTI) and dynamic susceptibility contrast (DSC) perfusion sequences. Scans were acquired across multiple 1.5 T and 3.0 T scanners from different manufacturers, as detailed in the accompanying acquisition parameter files. All images were rigidly co-registered, resampled to 1 mm³ isotropic resolution, skull-stripped using BrainMaGe, and defaced for privacy. Tumor annotations were generated using an ensemble of state-of-the-art deep learning models (DeepMedic, DeepSCAN, nnU-Net) fused via STAPLE, with manual refinement on a subset of cases by expert neuroradiologists. The final segmentations delineate three subregions: enhancing tumor, non-enhancing or necrotic core, and peritumoral edema or infiltration.

### Descriptive statistics

Demographic data of the participants are contained in **Table 1**.

**Table 1:** Descriptive statistics.

Characteristic	Total (N = 661)	UCSF (N = 399)	UPENN (N = 262)
Age, (mean, SD)	60.9 (13.3)	59.5 (13.8)	63.0 (12.2)
Sex, n (%)			
Male	412 (61.6%)	248 (60.9%)	164 (62.6%)
Female	257 (38.4%)	159 (39.1%)	98 (37.4%)
Grade			
3	22 (3.3%)	22 (5.5%)	0 (0.0%)
4	639 (96.7%)	377 (94.5%)	262 (100.0%)
Methylated MGMT, n (%)	406 (60.8%)	295 (72.7%)	111 (42.4%)
Mutated IDH, n (%)	47 (7.0%)	40 (9.8%)	7 (2.7%)

### Radiomics extraction

For each patient of the UCSF-PDGM dataset, the available modalities comprised ADC, ASL, DWI, FLAIR, T1, T1-contrast, and T2, whereas for UPEN-GBM patients, only FLAIR, T1, T1-contrast, and T2 were available. Before radiomic feature extraction, all modalities except ADC, ASL, and DWI were subjected to N4 bias-field correction, and ensured to be isotropically spaced, to 1mm × 1mm × 1mm =*, normalizeScale=100, and binwidth=25*. Advanced MRI modalities in both datasets were provided as pre-processed 3D quantitative volumes rather than raw 4D acquisitions. In UCSF-PDGM, ASL appears as a single perfusion-weighted 3D map, and diffusion information is supplied as a 3D ADC volume. No 4D DSC data or multi-b-value DWI suitable for kinetic modelling were available. These images were therefore processed in the same manner as the structural modalities, namely converted to isotropic 1-mm grids, underwent no N4 correction, resampled to the tumor-mask space, and z-scored within the mask prior to radiomic feature extraction. It should be noted that since both the external and PyRadiomics normalizations use tumor-region statistics, the second normalization step effectively only multiplies the already normalized voxels by a factor of 100, without further altering relative grey-level patterns. Feature extraction was performed on the original volumes of all available modalities and versions of those subjected to Laplacian-of-Gaussian (σ=0.5, 1.0, 2.0, 3.0) and 3D Wavelet transformations. The resulting radiomic features encompassed multiple families describing complementary aspects of the tumor signal and geometry:

- 3D shape descriptors, computed on the original volumes, include volume, surface area, compactness, and sphericity.
- First-order statistics, capturing the intensity distribution (mean, median, energy, entropy, skewness, kurtosis, and percentile-based metrics).
- Gray-Level Co-Occurrence Matrix (GLCM) textures characterize the spatial co-occurrence of intensity pairs (contrast, correlation, homogeneity, angular second moment/energy, entropy, and more).
- Gray-Level Run-Length Matrix (GLRLM) textures, describing consecutive runs of equal gray levels (short/long run emphasis, gray-level non-uniformity, run non-uniformity, and more).
- Gray-Level Size-Zone Matrix (GLSZM) textures, quantifying zones of connected voxels with the same gray level (small/large zone emphasis, zone non-uniformity, zone entropy, and more).
- Gray-Level Dependence Matrix (GLDM) textures, assessing local gray-level dependencies (dependence entropy/variance, low/high gray-level emphasis, and more).
- Neighborhood Gray-Tone Difference Matrix (NGTDM) textures measure intensity differences between a voxel and its neighborhood (coarseness, contrast, busyness, complexity, strength).

### Radiomic features importance ranking

The extracted features were split into a training and Cross-Validation (train-CV) dataset containing 85% of the data, and a held-out test set with the remaining 15%, for objectivity in metric reporting. To avoid leakage and simultaneously maintain a balance between samples with methylated (class-1) and unmethylated (class-0) MGMT, the test set consisted of samples from patients that were not included in the train-CV set, while preserving the class ratio as much as possible. Those splits were saved before ranking and used in some performance benchmarking models.

For the train-CV set, a pipeline was built to determine relative feature importance. It consisted of a variance cutoff to exclude features that had variance less than 10^-8^, followed by a Spearman correlation filter with a threshold of 0.80, which dropped one feature from every pair whose absolute Spearman correlation exceeded 0.80. Next, features were z-scored before passing through a sparse Logistic Regressor. The latter step aimed to fit the train-CV data on the regressor and extract the appointed coefficients of those features, as a measure of relative importance.

For consistency, a permutation analysis was performed to evaluate the deterioration in model prediction if information in one feature is destroyed. Firstly, for each of the 5 splits, AUC and log-loss were computed on the validation fold. Log-loss measures how well calibrated the probabilities are, and is defined in Equation 1, where $y_{i}\in \{0,\;1\}$ are the class labels and $p_{i}\in [0,\;1]$ are the predicted class-1 probabilities. These metrics serve as a baseline for the next step and inform about the model’s true performance on the validation fold before any perturbation. Secondly, each feature that survived the variance and correlation filters gets mixed with all samples during the perturbation step. This retains the exact feature distribution but removes the relationship with the target class (methylated or unmethylated MGMT). Then, the new log-loss is computed on the latest predictions, and the increase compared to the baseline value of the intact folds is evaluated as a quantification of the performance deterioration. The shuffling procedure is repeated 100 times for each feature to eliminate randomness and acquire a stable importance score. Each feature’s relative importance is ranked by ordering the features by the average differences in log-loss from the baseline value. Finally, the metric that determines the importance ranking is a custom consensus rank, which is defined as the sum of the ranks corresponding to the Logistic Regressor coefficients and the ranks corresponding to the permutation importance. The 500 most important ranked features are then saved separately for training some of the models.

Notably, UPENN-GBM samples do not have ASL, ADC, and DWI modalities, so the corresponding radiomic features do not exist, as in the UCSF-PDGM dataset containing those modalities. The pipeline has been configured to manage missing features in samples, thus taking advantage of the extra modalities whenever available, or ignoring their corresponding radiomic features if missing. This effect is achieved by imputing missing values in UPENN-GBM samples with medians computed during training from UCSF-PDGM values. These values are constant for all UPENN-GBM samples; thus, the models that follow the pipeline learn to effectively ignore the imputed features by identifying the imputation constants. Alternatively, in UCSF-PDGM samples, the models utilize the information in those features, as their values differ from the imputation median value, which they have learned to recognize and ignore during training.

(1) $$LogLoss=-\frac{1}{N}\sum_{i=1}^{N}[y_{i}\log(p_{i})+(1-y_{i})\log(1-p_{i})]$$

### Model hyperparameter optimization and training

Six different model builds are tested to determine the best training strategy. Half are based on Logistic Regression, and the other is based on the Light Gradient Boosting Machine (LightGBM) architecture. For each model type, one model is trained on the train-CV set containing all the radiomic features, another on the train-CV set that retains the 500 most important features, and a third one on the original train-CV set but with an L1-regularized logistic regressor preceding the base architectures of each model type. The latter applies a logistic regression-based filter that removes a customizable percentage of redundant features based on their coefficients. It utilizes an extra term to the loss apart from the classic log-loss, leading to a mixed loss value defined in Equation 2. This term encourages the optimizer to reduce the weight values already very low to exactly zero, thereby suppressing redundant features and filtering noise. For each of the six builds, the hyperparameters are optimized using Optuna (14), with 400 trials. This step ensures that the highest capabilities of each build are revealed by selecting high-performing combinations of hyperparameters, such as tree depth, learning rate, regularization parameters, solver types, penalty types, and, in the case of the L1-filtered builds, the percentage of filtered features. The criterion Optuna considers for quantifying performance is the mean AUC across train-CV folds, ensuring no leakage from the test set.

### Probability calibration

Classification models often produce miscalibrated probabilities, meaning that their output scores do not always reflect the true likelihood of class 1, particularly under class imbalance or limited training data. For example, a predicted probability of 0.7 does not necessarily correspond to a 70% chance that the sample belongs to the positive class. This may cause the optimal decision threshold to deviate substantially from 0.5, leading to false clinical interpretations. Platt scaling (logistic calibration) was applied to the validation fold predictions, fitting a logistic regression model that maps raw classifier scores to calibrated probabilities to correct this. This procedure adjusts the probability scale without altering the model’s discrimination, thus retaining the same AUC score. After calibration, the decision threshold was determined by minimizing the sum of false positives and false negatives on the validation set. Calibration quality was quantified using the Brier score, which measures the mean squared error between predicted probabilities and true binary labels. A Brier score of 0.0 indicates perfect calibration, whereas higher values correspond to unreliable probability estimates.

### Decision curve analysis

Clinical utility of the highest-performing model was assessed on the test set using Decision Curve Analysis, which quantifies the net clinical value of model-assisted clinical decisions by balancing the benefits of true-positive predictions against the harms of false positives (15). This analysis allows identification of the range of threshold probabilities where the model provides a higher net benefit than the default strategies of treating all or none. In practice, this indicates the probability range within which using the model to guide decisions would lead to improved patient outcomes or fewer unnecessary interventions compared with the naive approaches. This clinical setting refers to whatever clinical action would be taken if a patient is predicted to have a methylated MGMT gene.

Net Benefit (NB), defined in Equation 2, quantifies the additional number of true positives (TP) achieved by the model, after accounting for the relative harm associated with false positive decisions (FP), at a given probability threshold ( $p_{t}$). From this stems the Net Reduction in unnecessary Interventions (NRI), defined in Equation 3, which measures how many unnecessary treatments or tests were avoided per 100 patients by using the model instead of treating every patient.

(2) $$NB=\frac{TP}{N}-\frac{FP}{N}\times \frac{p_{t}}{1-p_{t}}$$

(3) $$NRI=(NB_{model}-NB_{treat-all})\times \frac{1-p_{t}}{p_{t}}\times 100$$

### Explainability

Shapley values were used to quantify the contribution of each feature to the model’s output prediction. SHAP assigns an additive attribution score to every feature, ensuring that the sum of all feature attributions equals the predicted probability for a given sample. Positive Shapley values indicate that a feature increases the likelihood of the methylated MGMT (class 1) label, whereas negative values push the prediction towards the unmethylated MGMT (class 0) label. For each sample x, the model output f(x) can be decomposed as the sum of individual feature contributions $\varphi_{j}$, and a baseline value $\varphi_{0}$ represents the model’s expected output over the background (training) data distribution. This additive formulation is expressed in Equation 4, where $N_{f}$ denotes the total number of input features. The base value $\varphi_{0}$ corresponds to the average model prediction before observing any specific feature values, while each $\varphi_{j}$ quantifies the marginal contribution of a feature j to the final prediction.

(4) $$f(x)=\varphi_{0}+\sum_{j=1}^{N_{f}}\varphi_{j}$$

Along with Shapley values, the explainability of the proposed approach is enhanced by the overview of the consensus rank determined before training, as well as the separate logistic regression weights and permutation ranks that comprise it.

## Results

### Radiomic features importance ranking

Feature importance was determined using the top 500 features retained after ranking. Wavelet contributes 364/500 (72.8%), LoG 119/500 (23.8%), and Original 17/500 (3.4%). Within wavelets, sub-bands are broadly represented, with LLL most frequent (63), followed by LLH/HHH (46 each), LHL (45), HLL (44), LHH (42), and HLH/HHL (39 each); sub-bands containing ≥2 low-pass components (LLL, LLH, LHL) account for 154/364 (42.3%), indicating a modest preference for coarser spatial content under the consensus criterion. For LoG, larger kernels dominate: σ=3.0 mm (49/119) > 1.0 mm (29/119) > 0.5 mm (24/119) > 2.0 mm (17/119).

Modalities contribute balanced signal: T1 (88/500, 17.6%), ADC (77/500, 15.4%), FLAIR (71/500, 14.2%), T2 (70/500, 14.0%), DWI (68/500, 13.6%), ASL (66/500, 13.2%), and T1-ce (60/500, 12.0%). By family, GLSZM (141/500, 28.2%), GLCM (128/500, 25.6%), and First-order (101/500, 20.2%) dominate, with NGTDM (62/500, 12.4%) and GLDM (41/500, 8.2%) contributing less, and GLRLM (24/500, 4.8%) and Shape (3/500, 0.6%) rare - consistent with redundancy filtering minimizing purely morphological descriptors. Within First-order, Skewness (25), Kurtosis (20), Median (19), and Mean (18) are most frequent, highlighting distribution-shape statistics at filtered scales. GLSZM emphasizes Small Area Emphasis (43), Small Area Low Gray Level Emphasis (28), and Zone Entropy (22), i.e., small-zone heterogeneity markers. GLCM is led by MCC (31), Cluster Shade (29), Inverse Variance (15), IMC1 (12), and Correlation (11), reflecting second-order structural dependencies beyond simple contrast. NGTDM contributes Contrast (25), Strength (21), and Complexity (13); GLDM is dominated by Dependence Variance (11) with smaller contributions from DNU-normalized (9). Overall, the consensus indicates the classifier relies primarily on filtered, texture-centric cues at coarser spatial scales, with balanced modality support and minimal reliance on unfiltered or shape features.

**Figure 1:**
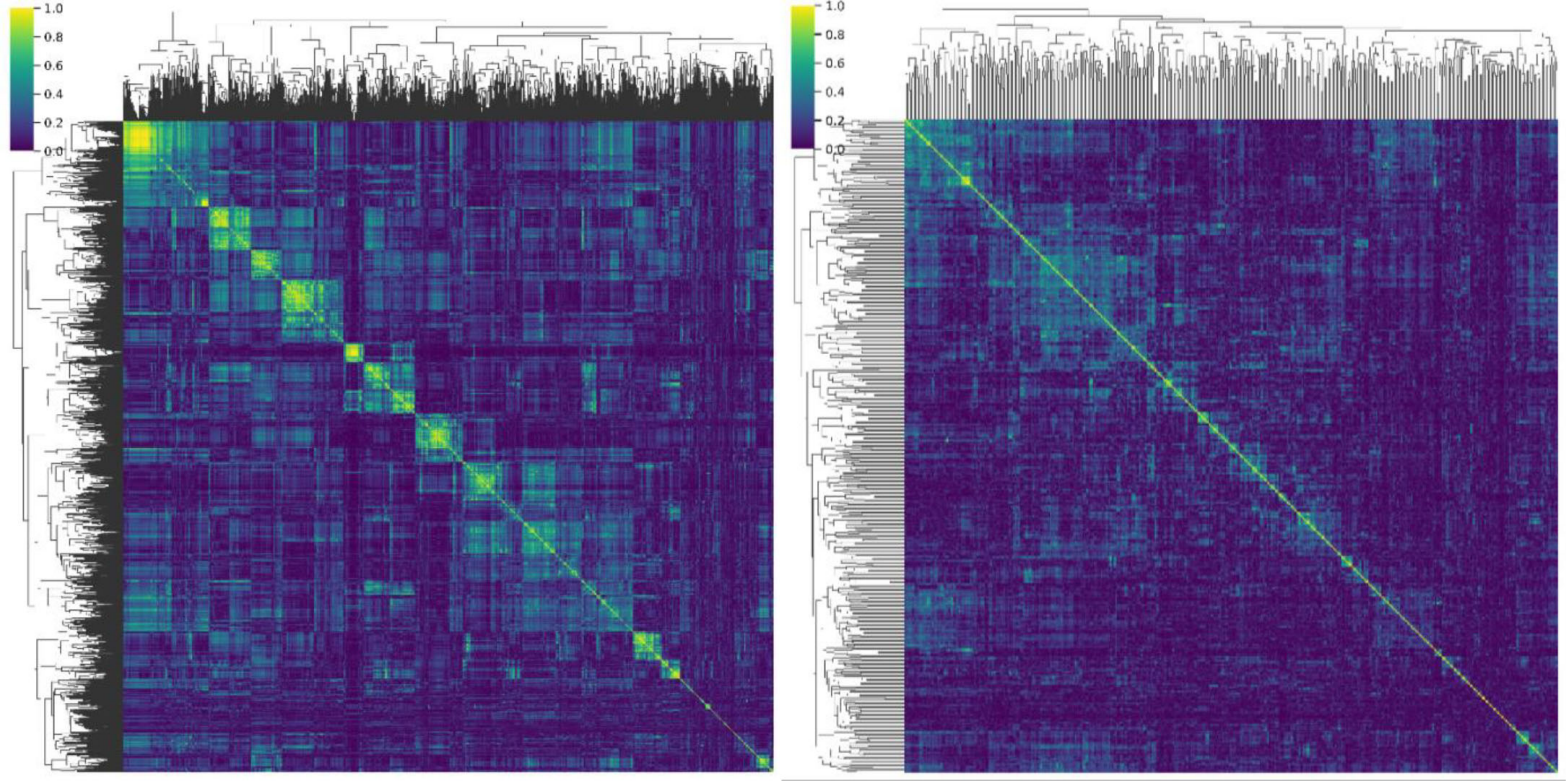
[Left] Pair-wise correlation matrix between all features, before filtering. [Right] Post-filtering correlation matrix between the 500 most indicative features for prediction of MGMT methylation status. Brighter spots correspond to highly correlated features, and darker spots correspond to less to no correlation. The right-hand-side matrix is overall darker, indicative reduced redundancy.

**Figure 2:**
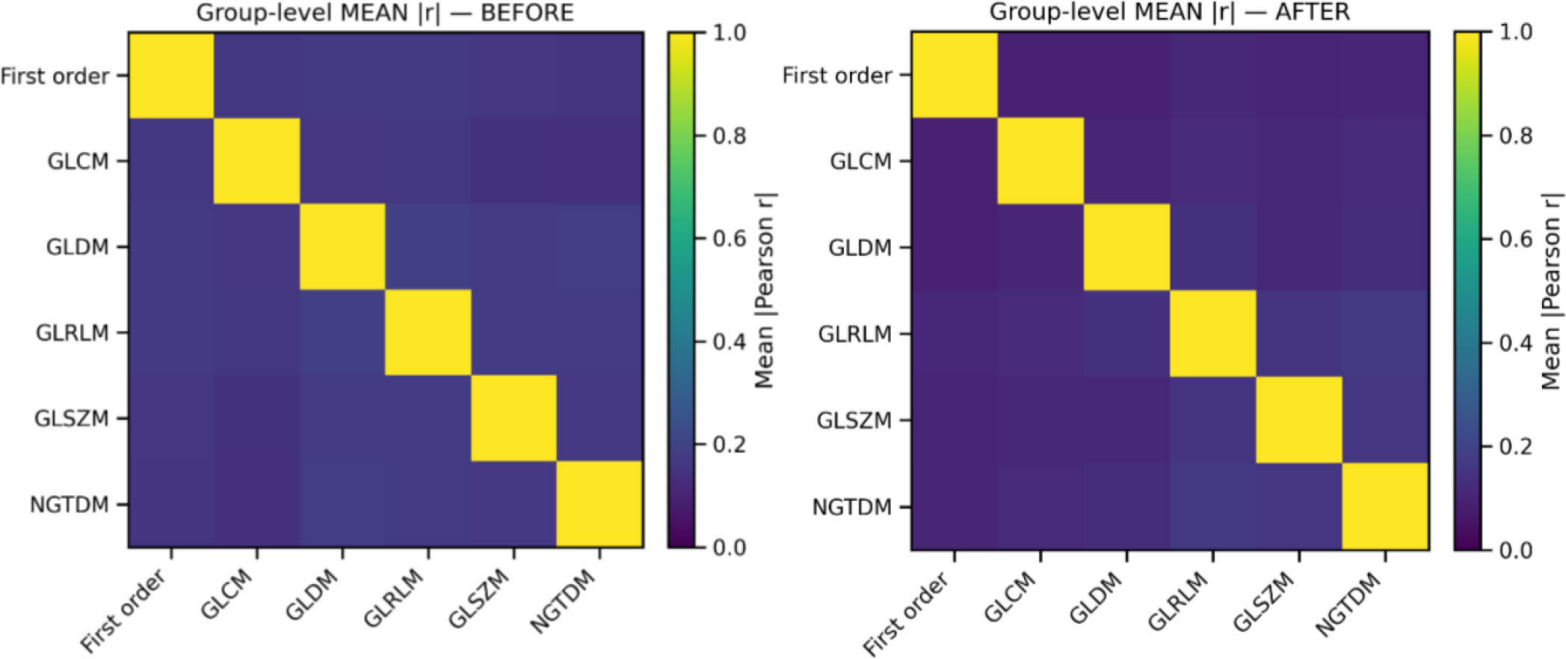
Average correlation between features of each group before (left) and after (right) filtering for redundancy reduction. The right-hand-side matrix is mostly dark violet, contrary to the left-hand side matrix, which appears blue, which, according to the colorbar, indicates that features are much less correlated and signal has been retained while redundant features have been excluded.

**Figure 3:**
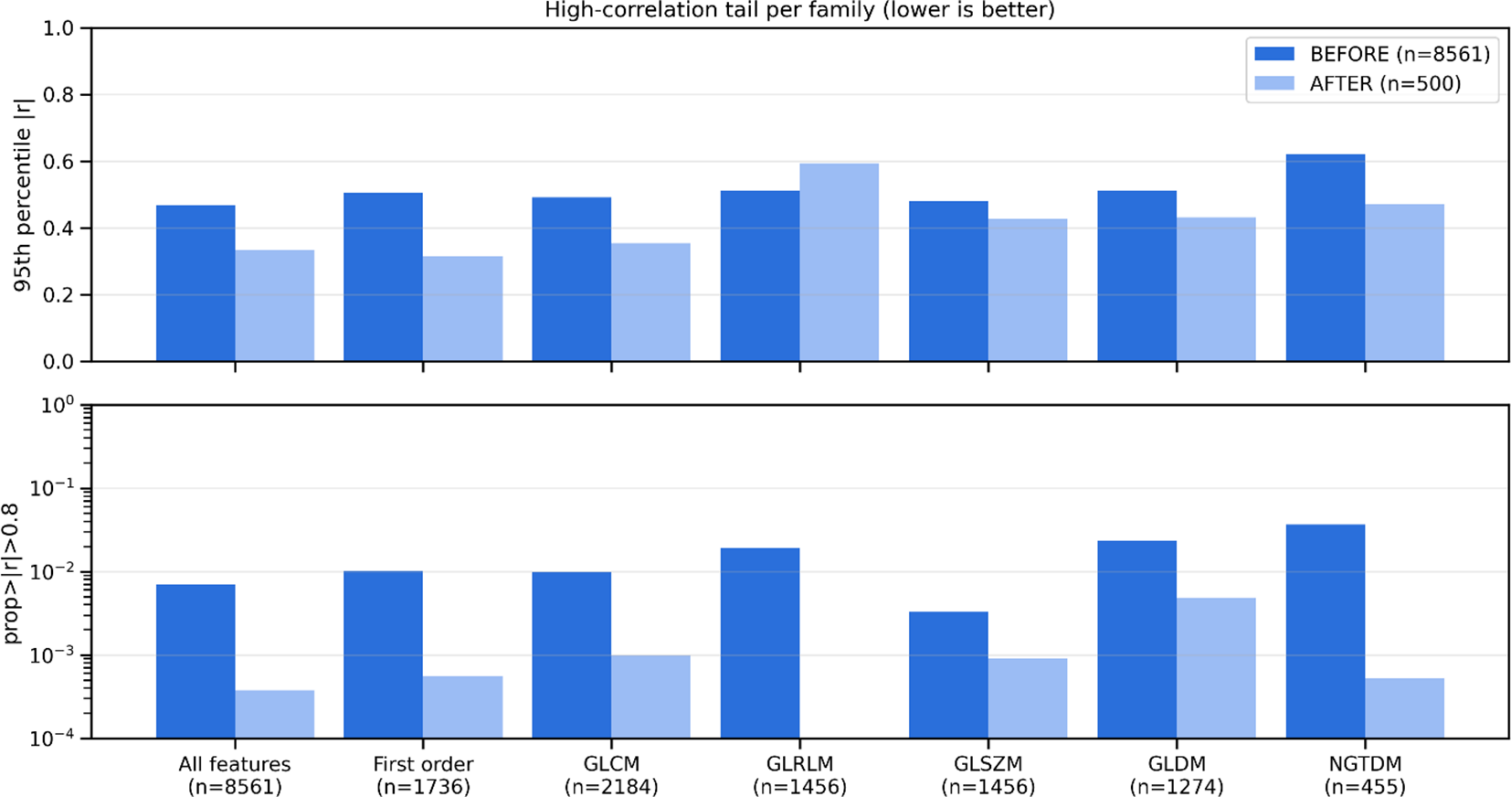
[Top] The 95^th^ percentile of pairwise Pearson correlation absolute values (|r|), where taller bars indicate stronger correlation and higher redundancy. [Bottom] The proportion of feature pairs with |r|>0.8, where taller bars indicate more highly redundant pairs. After filtering, Lower bar heights indicate that extreme correlations’ strength and frequency are reduced, translating to less feature redundancy.

**Figure 4:**
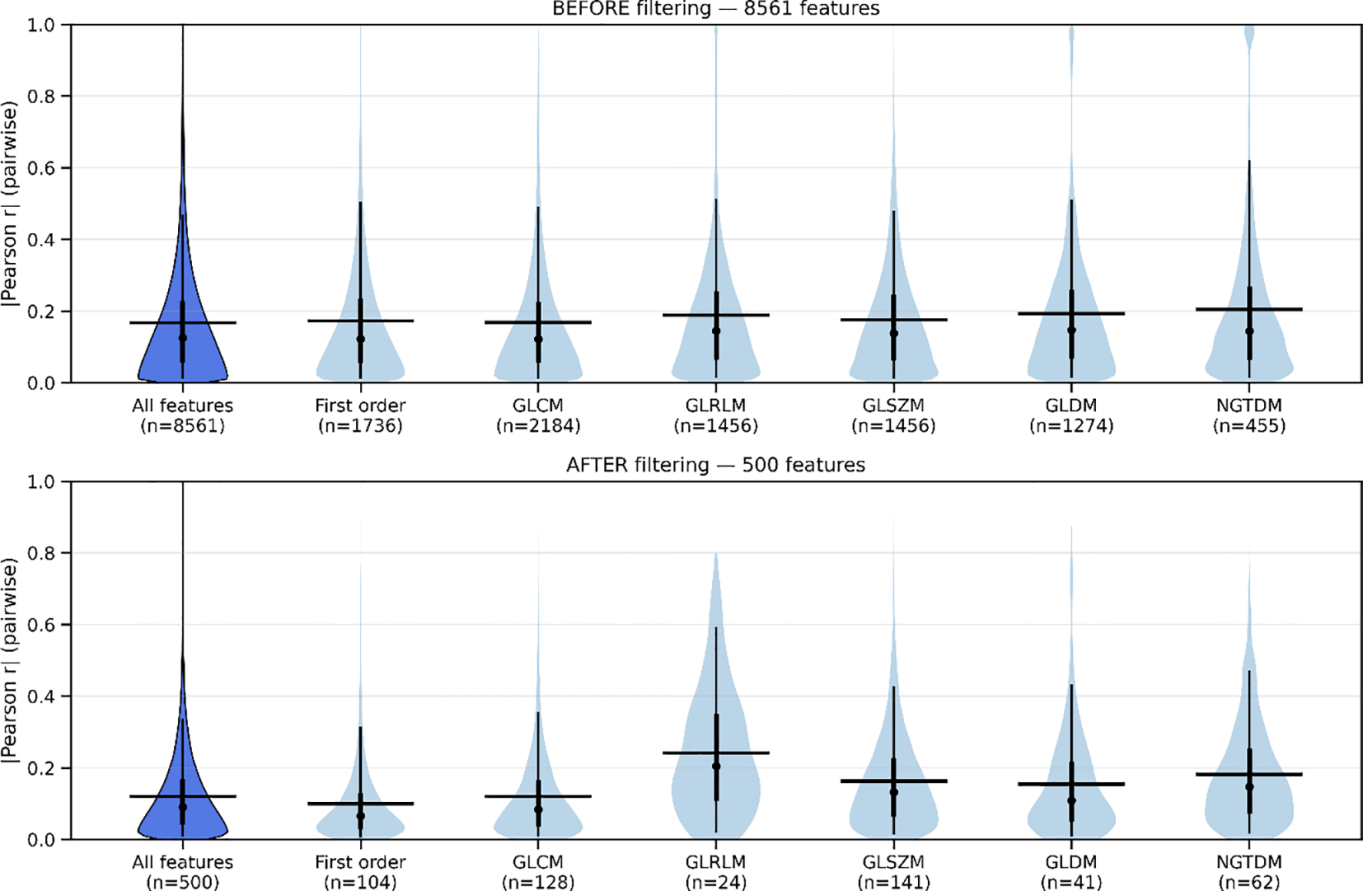
[Top] Violin plots of pairwise absolute Pearson correlation values (|r|) for all and per-family radiomic features before filtering, showing the distribution and spread of inter-feature dependencies. [Bottom] The same distributions after filtering, where narrower and lower violins indicate reduced correlation strength and variability. The visible contraction of the distributions reflects the effective removal of highly redundant features and improved independence among retained descriptors.

The ranking procedure reduced redundancy by removing highly correlated features, minimizing noise while retaining the useful signal. **Figure 1** presents the pair-wise feature correlation matrices before and after filtering. Bright yellow elements indicate high absolute Pearson correlation (|r| ≈ 1.0), whereas dark blue indicates little to no correlation (|r| ≈ 0.0). The visible reduction in bright regions after processing demonstrates substantially lower inter-feature correlation. Moreover, **Figure 2** summarizes correlations for radiomic features within the same families, showing generally dark violet blocks after filtering, contrary to blue ones before filtering, consistent with decreased redundancy within radiomic groups, according to the figure’s colorbar. **Figure 3** reports the distribution of pairwise correlations by family. The top panel shows the 95th percentile of |r|, and the bottom panel shows the fraction of pairs exceeding a high-correlation threshold (|r| > 0.8). Across most families, filtering attenuates the upper tail of the correlation distribution. Notably, the 95th percentile |r| declines for the overall feature set, GLCM, and GLSZM, indicating effective removal of highly redundant descriptors, as a lower 95th percentile implies less extreme pairwise redundancy and fewer near-duplicates. The improvement is even more pronounced in the proportion of strongly correlated pairs, which drops by approximately an order of magnitude in several families, like First order, GLCM, and GLSZM. **Figure 4** complements this analysis by presenting summary statistics of the correlation distributions. The top panel shows the 95th percentile of |r|, while the bottom panel reports the proportion of feature pairs with |r| > 0.8 on a logarithmic scale. These results confirm that correlation filtering yields a more compact, less collinear subset of radiomic features while preserving meaningful feature diversity and limiting unnecessary noise during model training.

### Classification

**Figure 5:**
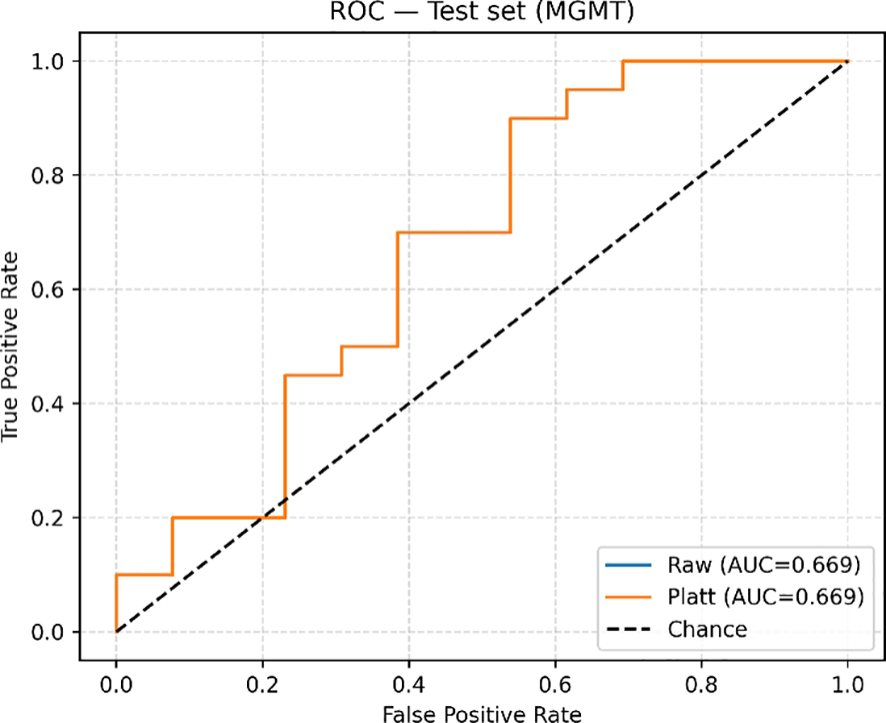
ROC-AUC curve upon evaluation on the test set.

Classification metrics for each of the six builds are summarized in **Table 2**. The average validation fold and the test set metrics are reported for clarity. The best-performing model is the LightGBM, trained on the 500 most important features, with an AUC of 0.67 and a recall of 0.90, reported on the held-out test set (**Figure 5**). Notably, it is closely followed by the L1-Filtered LightGBM, which indicates that strong filtering made a difference in model performance. The same trend can be observed in the Logistic regression models, as the AUC reported is significantly larger in the models trained with filtered features compared to those trained on all of them. In most builds, recall was higher than accuracy, which indicates that they have increased capabilities of avoiding false negatives, i.e., misdiagnosis of methylated MGMT.

**Table 2:** Classification metrics for the six builds, reported for the final validation fold and the test set.

Architecture		Logistic regression			LightGBM		
Features included	Metric	All	Top 500	L1-filtered	All	Top 500	L1-filtered
Final Validation Fold	AUC	0.68	0.76	0.71	0.66	**0.66**	0.67
	Recall	0.82	0.84	0.85	0.67	**0.71**	0.85
	Accuracy	0.67	0.70	0.67	0.65	**0.66**	0.67
	Precision	0.69	0.72	0.68	0.69	**0.72**	0.69
	Specificity	0.44	0.50	0.38	0.47	**0.57**	0.40
	F1-score	0.75	0.78	0.76	0.73	**0.71**	0.77
Test Set	AUC	0.55	0.65	0.66	0.65	**0.67**	0.62
	Recall	0.85	0.56	0.85	0.85	**0.90**	0.90
	Accuracy	0.61	0.64	0.64	0.70	**0.72**	0.70
	Precision	0.63	0.67	0.65	0.71	**0.72**	0.69
	Specificity	0.23	0.38	0.31	0.46	**0.46**	0.38
	F1-score	0.72	0.73	0.73	0.77	**0.80**	0.78

Bold values indicate the highest performance achieved for each metric across feature-selection strategies within the same classifier.

### Decision curve analysis

**Figure 6:**
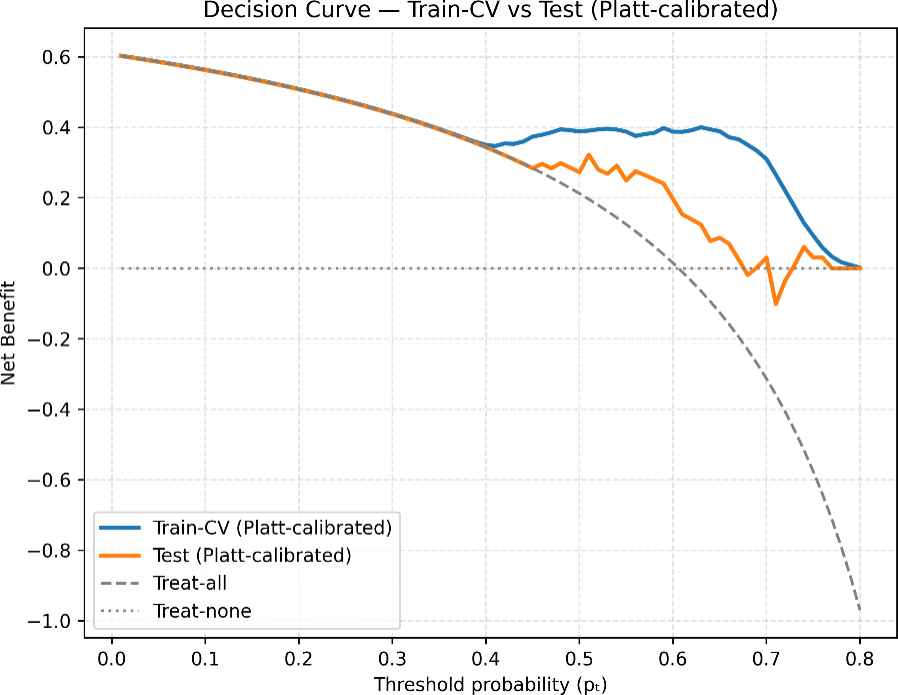
Decision curve analysis comparing train-CV and test cohorts for the Platt-calibrated, highest-performing model.

The clinical utility of the highest-performing model was assessed using Decision Curve Analysis (DCA) to evaluate the clinical value of model-assisted decisions across a range of decision thresholds ( $p_{t}$). In the test set, before probability calibration, the model achieved a maximum Net Benefit (NB) of 0.143 at the operating threshold $t^{*}=0.575$, corresponding to a Net Reduction in unnecessary Interventions value of NIR_100_ = 4.5 per 100 patients compared with the naive strategy of treating all patients. After Platt calibration, the model’s decision curve improved, achieving a notably higher NB = 0.264 at the same threshold, equivalent to avoiding approximately NIR_100_ = 13.6 unnecessary interventions per 100 patients. Across the clinically relevant range of $p_{t}=0.35$ to 0.65, the calibrated model consistently outperformed both treat-everyone and treat-none strategies, indicating that using its predicted probabilities to guide decisions could provide a meaningful clinical advantage. A comparison between train-CV and test set DCAs, demonstrated consistent curve shapes and overlapping beneficial ranges, confirming that the model’s decision utility generalizes well beyond the training data, as seen in **Figure 6**. In both sets, calibration improved the model’s net benefit, particularly at intermediate threshold probabilities where clinical uncertainty tends to be greater. Specifically, model interpretation for each output interval is shown in **Table 3**. These results suggest that the proposed model could support individualized decision-making by improving the balance between true-positive identification and avoiding unnecessary clinical interventions.

**Table 3:** Model interpretation for different output probabilities.

Predicted probability range (Platt calibrated)	Model interpretation	Clinical implication
$0\leq p_{cal}<0.300$	Assume unmethylated MGMT with high confidence	Suggests limited expected benefit from temozolomide. Prioritize radiotherapy, immunotherapy, while always confirming MGMT status through histopathology/molecular testing before final therapeutic decisions.
$0.300\leq p_{cal}<0.575$	Low confidence of MGMT methylation status; Strongly consider confirmatory testing	Proceed with biopsy or resection for definitive MGMT testing before planning therapy.
$0.575\leq p_{cal}<0.800$	Assume methylated MGMT with high confidence	May support early consideration of temozolomide-based therapy, pending molecular confirmation. Useful for preliminary stratification or surgical planning discussions.
$0.800\leq p_{cal}\leq 1.000$	Assume methylated MGMT with very high confidence	Can inform multidisciplinary discussions and early treatment planning while awaiting tissue-based confirmation.

### Explainability

**Figure 7:**
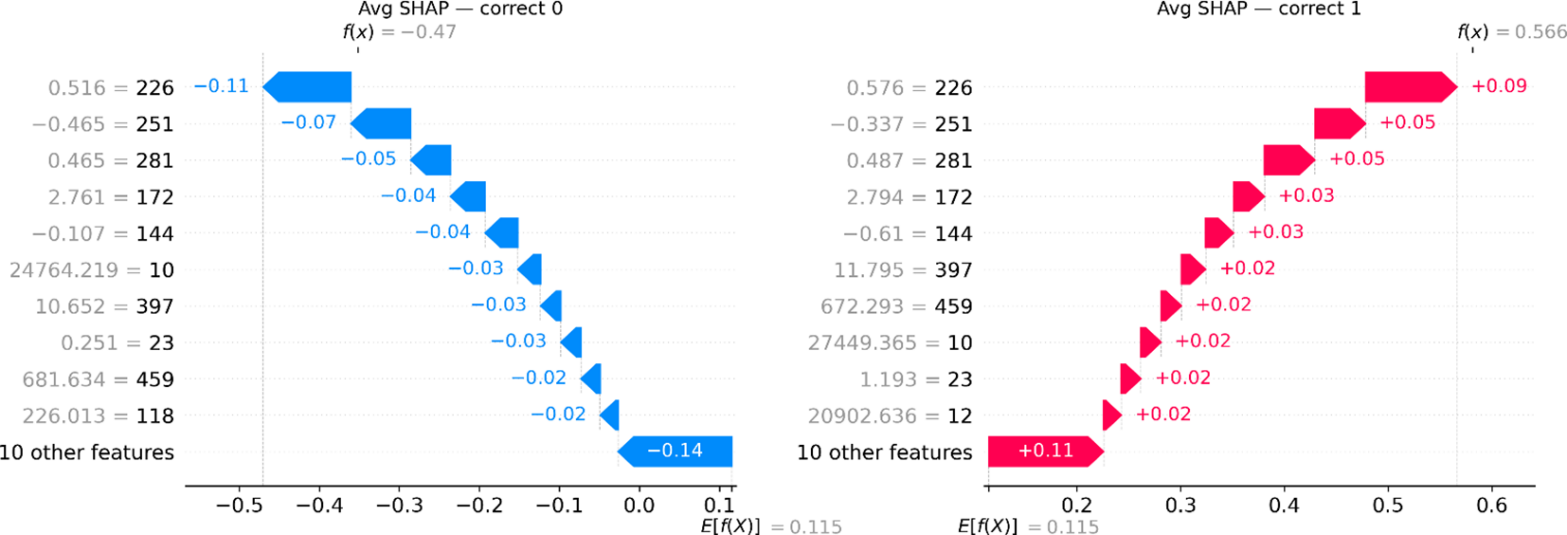
The average Shapley value diagrams illustrate the top 20 most influential features for the most confidently correctly classified class-0 sample (left) and the most confident correctly classified class-1 sample (right). The color and length of each arrow indicate the direction and magnitude of each feature’s influence in the classification decision. Red signifies influence towards the positive class and blue towards the negative class. E[f(x)] = 0.115 indicates the expected Shapley value (a neutral baseline). All features’ contributions are algebraically summed for the final Shapley value, f(x), to be produced (class-0 case: f(x) = -0.470,3 class-1 case: f(x) = 0.566).

**Figure 7** shows the ranking codes corresponding to the 20 most informative radiomic features, plotted against their respective Shapley values, for all correctly classified class-0 (left-side) and class-1 (right-side) samples of the entire dataset (train-CV and test set). **Table 4** shows the names corresponding to the displayed codes for **Table 4**.

**Table 4:** Index-feature name map.

Index	Modality	Filter	Family	Feature
226	T2	log sigma 0–5 mm 3D	GLSZM	SmallAreaEmphasis
251	ADC	wavelet LLH	FIRSTORDER	Median
281	T2	wavelet HLH	GLCM	InverseVariance
172	DWI	wavelet LHH	GLRLM	RunVariance
144	ADC	wavelet HHH	FIRSTORDER	Skewness
10	ASL	wavelet LLL	GLDM	LargeDependenceHighGrayLevelEmphasis
397	FLAIR	wavelet HLL	FIRSTORDER	Kurtosis
23	ADC	wavelet HLH	FIRSTORDER	Skewness
459	ADC	wavelet LLL	FIRSTORDER	RootMeanSquared
118	FLAIR	wavelet LLL	FIRSTORDER	Minimum
12	T2	wavelet LLL	GLSZM	ZoneVariance

## Discussion

In this study, we developed an explainable multicentric multimodal radiomics machine learning model for non-invasive MGMT status prediction in HGG using preoperative MRI. Based on Light GBM, the model achieved an AUC of 0.67 and a recall of 0.90 on the test set, demonstrating its effectiveness in accurately determining MGMT status and reducing false negatives, key factors for a predictive biomarker test. Probability calibration can further enhance classification accuracy and clinical utility. The modality adaptation strategy allows the model to use conventional sequences (T1, T2, FLAIR) and extend to advanced sequences (ADC/DWI, ASL) when available, making it suitable for different clinical settings where protocols vary. Additionally, implementing DCA and SHAP explainability guides clinicians’ next steps, reduces distrust in black-box AI, and supports non-invasive biopsy prediction, case discussions during tumor boards, and preliminary treatment planning while awaiting biopsy confirmation. Although the model’s limited ability to distinguish between positive and negative MGMT cases, as indicated by the AUC, shouldn’t replace tissue-based MGMT testing, it can help with earlier risk stratification before biopsy or surgery. It showed very high sensitivity, reducing the risk of missing methylated cases, which is crucial for temozolomide treatment. The calibrated decision curve demonstrated a positive net benefit across relevant thresholds, offering more useful information than standard methods. Despite a moderate AUC, its high sensitivity, solid calibration, and favorable decision value suggest it can support preoperative risk assessment and tumor board discussions while waiting for molecular results. Our results align with prior radiomics studies predicting MGMT promoter methylation from MRI. Korfiatis et al. (2016) using T2 texture-based features achieved an AUC of 0.85 with an SVM classifier, and Han et al. (2018) reported an AUC of 0.91 by combining tumor location, necrosis, ADC, and rCBF (5, 6). Compared with these, our model’s performance was modest but used multicenter data and incorporated intratumoral and peritumoral regions, a combination shown by Yu et al. (2020) to enhance robustness in MGMT prediction (16). Our model extends reported literature, offering improved interpretability through SHAP-based feature transparency and clinical benefit assessment.

Feature-importance analysis showed that the model mainly depends on wavelet and Laplacian–of–Gaussian texture features, with little reliance on morphological descriptors. This indicates a sensitivity to tumor heterogeneity rather than shape (10, 17). Previously, Drabycz et al. (2010) studied the link between visually assessed MRI features and MGMT promoter methylation in GBM. They found that ring enhancement on contrast-enhanced T1-weighted images was significantly associated with the unmethylated MGMT status (P = 0.006). Still, the overall accuracy of visual classification was only 71%, highlighting the limited reliability of conventional imaging markers (18). Their research also showed that texture differences on T2-weighted images could distinguish methylated from unmethylated tumors, implying that microstructural heterogeneity carries essential biological information (5, 18). Consistent with these observations, the balanced importance across MRI modalities (ADC, T1, ASL, FLAIR, T2, and DWI) indicates the model’s ability to integrate structural and physiologic characteristics related to cellularity (ADC) and perfusion (ASL), key indicators of MGMT status. Notably, T1 CE features ranked lowest in importance, reinforcing that enhancement characteristics alone are not a reliable marker for MGMT methylation status (6, 19).

SHAP-based explainability confirmed these findings by showing that texture- and intensity-derived features, particularly, were the strongest positive contributors to predicting MGMT status. Methylated gliomas tend to be more structurally uniform, whereas unmethylated tumors show chaotic signal variations with rim enhancement and necrotic core, reflecting their biologically more aggressive nature (8, 20). Also, among the top features, FLAIR wavelet-LLL First-order Minimum (feature 118, **Table 4**) consistently exhibited negative SHAP values, indicating that lower minimum FLAIR intensities at coarse spatial scales were associated with unmethylated MGMT status. This aligns with the known imaging characteristic of unmethylated GBM, which frequently displays necrotic low-signal regions on FLAIR sequences (18, 21, 22). The model also included the peritumoral region, capturing microstructural and diffusion heterogeneity beyond the enhancing core, as radiomics models tend to perform better when intratumoral and peritumoral areas are combined compared to each alone (11). Previous research shows that peritumoral ADC and texture heterogeneity are linked to tumor infiltration and MGMT methylation status, as the invasive margin reflects both cellular diffusion restriction and vasogenic edema in HGG (16). Ladenhauf et al. (2023) reported that in 42 patients with GBM, unmethylated tumors had significantly higher ADC values compared to methylated ones (p = 0.002, p = 0.0007 for absolute and normalized values, respectively) (23). The unmethylated HGGs often exhibit more edema, lower ADC values, and increased perfusion metrics (19). Hence, features derived from ADC and ASL modalities had a decisive influence, aligning with biological expectations that methylated tumors display lower DWI, higher ADC, and reduced perfusion (ASL), due to decreased cellular density (6). These findings suggest the model has captured biologically meaningful patterns consistent with known MGMT-related imaging features, increasing confidence in its clinical relevance.

### Limitations

As expected, this study has limitations. The primary limitation is the absence of external validation, which restricts the model’s generalizability. An ideal external validation dataset should be multicentric, collected from different MRI scanners, vendors, and protocols, to ensure broad applicability. It should also include a balanced number of methylated and unmethylated MGMT cases, with consistent, high-quality segmentations of intratumoral and peritumoral regions for reliable radiomic analysis. To evaluate the model’s modality-adaptive design, it should incorporate the same set of conventional (T1, T2, FLAIR) and advanced MRI sequences (ADC/DWI and ASL). Including diverse patients would further test the model’s robustness to real-world variations, providing a more thorough assessment of its performance stability and clinical utility across various imaging centers.

Additionally, although our framework successfully integrates multimodal MRI sequences, further incorporation of molecular and clinical data could enhance predictive accuracy and provide a more comprehensive understanding of tumor biology. Future research should include external validation using diverse, multi-institutional datasets and detailed clinical variables, such as patient age, Karnofsky performance status, tumor grade, and treatment history. Including molecular biomarkers beyond MGMT, like IDH1/2 mutation status, ATRX loss, TERT promoter mutation, EGFR amplification, and 1p/19q co-deletion, can improve the model’s understanding of tumor biology. These inputs could lead to more precise, personalized predictions and greater generalizability within real-world clinical workflows.

## Conclusions

This study introduced a radiomics-based ML model for noninvasive prediction of MGMT promoter methylation in high-grade gliomas, using multi-institutional multiparametric MRI. The model revealed biologically meaningful links aligned with known imaging phenotypes by including intratumoral and peritumoral regions and utilizing SHAP-based explainability. These findings highlight radiomics’ ability to extract clinically relevant information from standard MRI sequences, providing a reproducible, transparent method for tumor biomarker analysis. Future research should focus on external validation and integrating clinical and molecular biomarker data (IDH mutation, ATRX loss) to improve the model’s generalizability and support its clinical application.

## Data Availability

The datasets presented in this study can be found in online repositories. The names of the repository/repositories and accession number(s) can be found below: UPENN GBM (DOI: 10.7937/TCIA.709X-DN49, https://www.cancerimagingarchive.net/collection/upenn-gbm/), UCSF-PDGM (DOI: 10.7937/tcia.bdgf-8v37, https://www.cancerimagingarchive.net/collection/ucsf-pdgm/).
